# Psychometric Properties of the Oral Health Values Scale and Cultural Adaptation in the Indian Population

**DOI:** 10.7759/cureus.53942

**Published:** 2024-02-09

**Authors:** Upendra S Bhadauria, Bharathi Purohit, Nilima Nilima, Harsh Priya, Waidya N Hansraj, Sarveshwari Singh

**Affiliations:** 1 Division of Public Health Dentistry, All India Institute of Medical Sciences, New Delhi, IND; 2 Department of Biostatistics, All India Institute of Medical Sciences, New Delhi, IND; 3 Department of Dentistry, All India Institute of Medical Sciences, New Delhi, IND; 4 Conservative Dentistry and Endodontics, Sardar Patel Postgraduate Institute of Dental and Medical Sciences, New Delhi, IND

**Keywords:** scale validation, oral health care, oral health, questionnaire development and validation, psychometric properties

## Abstract

Aim: The Oral Health Values Scale is a multidimensional instrument that identifies and measures a person's values related to oral health. This scale has been validated in American respondents. This study aims to evaluate the adaptability and validity of the Oral Health Values Scale in the Hindi language (H-OHVS).

Methodology: A total of 240 adults participated in the study that was carried out from July to November 2022. An offline one-to-one survey was carried out to collect data by an investigator blinded to the study protocol. The translation and testing of the OHVS were carried out based on the cross-cultural adaptation guidelines of the American Academy of Orthopedic Surgeons (AAOS) Evidence-Based Medicine Committee. The content validity was assessed by an expert panel. Construct validity was analyzed through Exploratory Factor Analysis (EFA), utilizing principal component analysis with varimax rotation. The internal consistency of the Hindi version of OHVS was evaluated using Cronbach’s alpha.

Results: The results showed that H-OHVS had an Item-level Construct Validity Index (I-CVI) ranging from 0.82 to 1.00. Two components, compliance and hesitance, were formed on H-OHVS accounting for 63.91% of the cumulative variance. The resulting model fit indices on confirmatory factor analysis presented an adequate fit to the data. The overall Cronbach's alpha coefficient for H-OHVS (α = 0.868) presents excellent internal consistency.

Conclusions: The study findings provide a certain degree of evidence in favor of this scale and establish the Oral Health Values Scale (OHVS) as a psychometrically sound measure.

## Introduction

Health behaviors play a crucial role in one's overall health, reflecting the importance an individual places on their well-being [1]. Oral health is a vital component of overall health and is closely linked to general well-being [2-3]. Oral health values are determined by various factors and interactions. Oral health values can be defined as the extent to which an individual views dental status as important, or their prioritization of and dedication to improving or maintaining their teeth, gingiva, and aspects of orofacial functioning [4].

Comparing it with other aspects of health, oral health values have been understudied and have not been assessed directly. The previous studies have primarily focused on a construct closely associated with the oral health values termed as the oral health-related quality of life. The various quality-of-life-related patient constructs have been criticized for reflecting the concerns of clinicians and researchers rather than what patients value [4-6]. 

To understand the beliefs about oral health values among adults, the Oral Health Value Scale (OHVS) was developed a year ago and was found to be reliable and valid [4]. The OHVS is a multidimensional instrument that identifies and measures the values of one’s oral health. During the study of development and validation, the OHVS was reported to exhibit good psychometric properties [4].

The value and importance of oral health in India, however, vary significantly from different parts of the world. The wide disparity in the provision of oral health services between rural and urban areas, along with significant variations in social parameters such as education and income within the population, further imposes barriers to the utilization of oral healthcare services [7].

Validity is not an inherent property of the measurement instrument; instead, it refers to the proposed interpretation and use of the instrument. Validity must be considered each time an instrument is used [8].

Understanding the oral health values of the Indian population in the Hindi language thus requires a culturally adapted and validated scale on oral health values. This study was thus carried out to adapt and validate the Oral Health Values Scale in Hindi language (H-OHVS) and evaluate the psychometrical properties of the scale.

## Materials and methods

The permission to conduct the study was taken from the institutional ethical committee of the institute. A cross-sectional descriptive study was carried out among 240 adult participants in both rural and urban field settings. The sample size estimation was conducted based on the principle that the ratio of the sample size to items assessed should be 1:10-1:20 [9-10], and a sample size of 240 was deemed appropriate. Adults aged 18 years and above, who provided informed consent, were included in the study. Adults with an inability to comprehend Hindi and cooperate with the study protocol were excluded from the analysis. The selection of experts was based on the following criteria: (1) experience of over five years in dental public health, oral health, and dentistry; (2) postgraduate and above degree and senior professional title; and (3) understanding of the Hindi language. The sociodemographic characteristics of the study participants were recorded using a self-designed proforma comprising name, gender, age, area of residence, and socioeconomic status.

Translation, adaptation, and psychometric testing

The scale was validated in American respondents and consisted of 12 items. Six of the 12 items on the scale are reversed, and the assessment is carried out on a continuum from 1 (strongly disagree) to 5 (strongly agree) [4]. The total score of the scale ranges from 12 to 60, taking into consideration four factors: Professional Dental Care and Cost, Appearance and Health, Flossing, and Retention of Natural Teeth [4]. The translation and testing of the OHVS was carried out based on the cross-cultural adaptation guidelines of the American Academy of Orthopedic Surgeons (AAOS) Evidence-Based Medicine Committee [11]. The steps from translation and testing comprised the following: forward translation, synthesis, backward translation, evaluation of content validity, and pre-experiment. In the forward translation step, two native bilingual researchers, one with a master's in Public Health Dentistry, experience of more than 10 years, and scholarly experience in the United States, and the other with a master's in the English language, independently translated the scale. In the synthesis step, a third native bilingual translator compared the two translated versions and formed the initial version of H-OHVS after discussion and confirmation among the research group members. Flossing as a measure of oral hygiene prevention is not a popular means of oral health maintenance in India. The domain toothbrushing was thus prioritized, and the component flossing was replaced by brushing. The initial version was back-translated into English by two researchers who were blinded to the original scale. The two back-translation versions were subsequently compared until a final Hindi-translated version was obtained. The evaluation of content validity was done by 11 experts who were consulted to integrate and culturally adjust H-OHVS. The revised H-OHVS was pilot-tested in 50 adults selected by convenience sampling. The assessment of whether the items could be easily understood and filled out was also conducted, following which the psychometric properties of the translated scale were assessed. Questionnaires (*n *= 240) were collected, and both exploratory factor and confirmatory factor analyses were carried out (Appendices A and B).

Data collection

The recruitment of the 240 adults was carried out from October 2023 to March 2023. Informed consent was obtained before the investigation. An offline one-to-one survey was carried out to collect data by an investigator blinded to the study protocol.

Statistical analysis

The data analysis was carried out using IBM SPSS Statistics for Windows, Version 23.0 (IBM Corp., Armonk, NY) software and Stata v.16.1 (StataCorp LLC, College Station, TX). Categorical variables were described using frequency and percentages. A *P *< 0.05 was considered statistically significant. The content validity was evaluated by an expert panel. Construct validity was analyzed by Exploratory Factor Analysis (EFA), using principal component analysis with varimax rotation. The internal consistency of H-OHVS was assessed using Cronbach's alpha. Confirmatory factor analysis (CFA) was performed using the structural equation model (SEM) to further evaluate the validity, adopting the root mean square error of approximation (RMSEA), Tucker-Lewis Index (TLI), comparative fit index (CFI), and standardized root-mean-square residual (SRMR). For this study, CFI > 0.90, TLI > 0.90, RMSEA < 0.08, and SRMR < 0.10 suggest an adequate fit. A Cronbach's alpha was used to summarize the internal consistency of the H-OHVS and its domains.

## Results

Sample characteristics

The average age of the study participants was found to be 31.24 ± 10.64 years. The descriptive characteristics of the sample are represented in Table 1. 

**Table 1:** Descriptive characteristics of the study sample.

Variable	Categories	*n* (%)
Gender	Male	169 (70.4)
	Female	71 (29.6)
Place of residence	Rural	76 (31.7)
	Urban	164 (68.3)
Educational qualification	Postgraduate/honors degree	47 (19.6)
	Graduate	90 (19.6)
	Intermediate/Diploma	61 (37.5)
	High school	32 (13.3)
	Middle school	6 (2.5)
	Primary school	3 (1.3)
	Illiterate	1 (0.4)
Occupation	Politician/senior official/manager	8 (3.3)
	Professional	61 (25.4)
	Technicians and support professionals	62 (25.8)
	Clerk	4 (1.7)
	Skilled workers, shop, and market sales professionals	26 (10.8)
	Skilled agricultural and fishery workers	20 (8.3)
	Crafts and related trade workers	2 (.8)
	Plant and machine operators and assemblers	15 (6.3)
	Primary business	30 (12.5)
	Unemployed	12 (5)
Total monthly income of the family (in rupees)	≥184,376	32 (13.3)
	92,191-184,370	26 (10.8)
	68,967-92,185	18 (7.5)
	46,095-68,961	42 (17.5)
	27,654-46,089	80 (33.3)
	9,232-27,648	42 (17.5)
	≤9,226	-

Content validity

A four-point Likert scale was utilized by the experts to evaluate the relevance of each item, ranging from not relevant to very relevant. The results showed that the H-OHVS had Item-level Construct Validity Index (I-CVI) ranging from 0.82 to 1.00.

Constructive validity and model fit

The correlation between different variables and the mean participant response to H-OHVS is represented in Tables 2-3. To investigate the theoretical constructs represented by the set of translated items under study, an exploratory factor analysis was performed. A Kaiser-Meyer-Olkin statistic (0.878) indicates excellent sampling adequacy. Bartlett's test of sphericity was found to be significant (*P *< 0.001), indicating that the H-OHVS has common factors, allowing us to proceed with dimension-reduction techniques.

**Table 2:** Correlation between different variables

		Q1	Q2	Q3	Q4	Q5	Q6	Q7	Q8	Q9	Q10	Q11	Q12
Q1	Pearson correlation	1	-0.150^*^	0.805^**^	-0.233^**^	0.807^**^	-0.169^**^	0.748^**^	-0.117	-0.094	0.768^**^	-0.246^**^	0.793^**^
	Sig. (two-tailed)		0.020	0.000	0.000	0.000	0.009	0.000	0.070	0.146	0.000	0.000	0.000
	N	240	240	240	240	240	240	240	240	240	240	240	240
Q2	Pearson correlation	-0.150^*^	1	-0.227^**^	0.454^**^	-0.116	0.380^**^	-0.214^**^	0.265^**^	0.193^**^	-0.122	0.226^**^	-0.148^*^
	Sig. (two-tailed)	0.020		0.000	0.000	0.073	0.000	0.001	0.000	0.003	0.059	0.000	0.022
	N	240	240	240	240	240	240	240	240	240	240	240	240
Q3	Pearson correlation	0.805^**^	-0.227^**^	1	-0.221^**^	0.819^**^	-0.150^*^	0.763^**^	-0.192^**^	-0.185^**^	0.775^**^	-0.185^**^	0.804^**^
	Sig. (two-tailed)	0.000	0.000		0.001	0.000	0.020	0.000	0.003	0.004	0.000	0.004	0.000
	N	240	240	240	240	240	240	240	240	240	240	240	240
Q4	Pearson correlation	-0.233^**^	0.454^**^	-0.221^**^	1	-0.160^*^	0.539^**^	-0.161^*^	0.339^**^	0.194^**^	-0.158^*^	0.399^**^	-0.132^*^
	Sig. (two-tailed)	0.000	0.000	0.001		0.013	0.000	0.012	0.000	0.003	0.015	0.000	0.041
	N	240	240	240	240	240	240	240	240	240	240	240	240
Q5	Pearson correlation	0.807^**^	-0.116	0.819^**^	-0.160^*^	1	-0.140^*^	0.760^**^	-0.139^*^	-0.186^**^	0.844^**^	-0.233^**^	0.875^**^
	Sig. (two-tailed)	0.000	0.073	0.000	0.013		0.030	0.000	0.032	0.004	0.000	0.000	0.000
	N	240	240	240	240	240	240	240	240	240	240	240	240
Q6	Pearson correlation	-0.169^**^	0.380^**^	-0.150^*^	0.539^**^	-0.140^*^	1	-0.177^**^	0.230^**^	0.387^**^	-0.141^*^	0.311^**^	-0.116
	Sig. (two-tailed)	0.009	0.000	0.020	0.000	0.030		0.006	0.000	0.000	0.029	0.000	0.072
	N	240	240	240	240	240	240	240	240	240	240	240	240
Q7	Pearson correlation	0.748^**^	-0.214^**^	0.763^**^	-0.161^*^	0.760^**^	-0.177^**^	1	-0.125	-0.071	0.800^**^	-0.109	0.840^**^
	Sig. (two-tailed)	0.000	0.001	0.000	0.012	0.000	0.006		0.052	0.270	0.000	0.091	0.000
	N	240	240	240	240	240	240	240	240	240	240	240	240
Q8	Pearson correlation	-0.117	0.265^**^	-0.192^**^	0.339^**^	-0.139^*^	0.230^**^	-0.125	1	0.263^**^	-0.155^*^	0.340^**^	-0.116
	Sig. (two-tailed)	0.070	0.000	0.003	0.000	0.032	0.000	0.052		0.000	0.016	0.000	0.072
	N	240	240	240	240	240	240	240	240	240	240	240	240
Q9	Pearson correlation	-0.094	0.193^**^	-0.185^**^	0.194^**^	-0.186^**^	0.387^**^	-0.071	0.263^**^	1	-0.156^*^	0.261^**^	-0.175^**^
	Sig. (two-tailed)	0.146	0.003	0.004	0.003	0.004	0.000	0.270	0.000		0.016	0.000	0.007
	N	240	240	240	240	240	240	240	240	240	240	240	240
Q10	Pearson correlation	0.768^**^	-0.122	0.775^**^	-0.158^*^	0.844^**^	-0.141^*^	0.800^**^	-0.155^*^	-0.156^*^	1	-0.238^**^	0.904^**^
	Sig. (two-tailed)	0.000	0.059	0.000	0.015	0.000	0.029	0.000	0.016	0.016		0.000	0.000
	N	240	240	240	240	240	240	240	240	240	240	240	240
Q11	Pearson correlation	-0.246^**^	0.226^**^	-0.185^**^	0.399^**^	-0.233^**^	0.311^**^	-0.109	0.340^**^	0.261^**^	-0.238^**^	1	-0.178^**^
	Sig. (two-tailed)	0.000	0.000	0.004	0.000	0.000	0.000	0.091	0.000	0.000	0.000		0.006
	N	240	240	240	240	240	240	240	240	240	240	240	240
Q12	Pearson correlation	0.793^**^	-0.148^*^	0.804^**^	-0.132^*^	0.875^**^	-0.116	0.840^**^	-0.116	-0.175^**^	0.904^**^	-0.178^**^	1
	Sig. (two-tailed)	0.000	0.022	0.000	0.041	0.000	0.072	0.000	0.072	0.007	0.000	0.006	
	N	240	240	240	240	240	240	240	240	240	240	240	240

**Table 3:** Mean participants responses to H-OHVS. Range of scores: 1-5. H-OHVS, Oral Health Values Scale in the Hindi language

	Mean	Standard deviation	Analysis, *N*
Q1	4.4125	1.19	240
Q2	4.0542	1.03	240
Q3	4.4042	1.16	240
Q4	4.0875	0.79001	240
Q5	4.4708	1.1199	240
Q6	4.2375	0.79607	240
Q7	4.2125	1.16462	240
Q8	3.7000	1.05180	240
Q9	3.8958	0.99032	240
Q10	4.4417	1.08467	240
Q11	3.2583	1.18918	240
Q12	4.4458	1.05754	240

**Figure 1:**
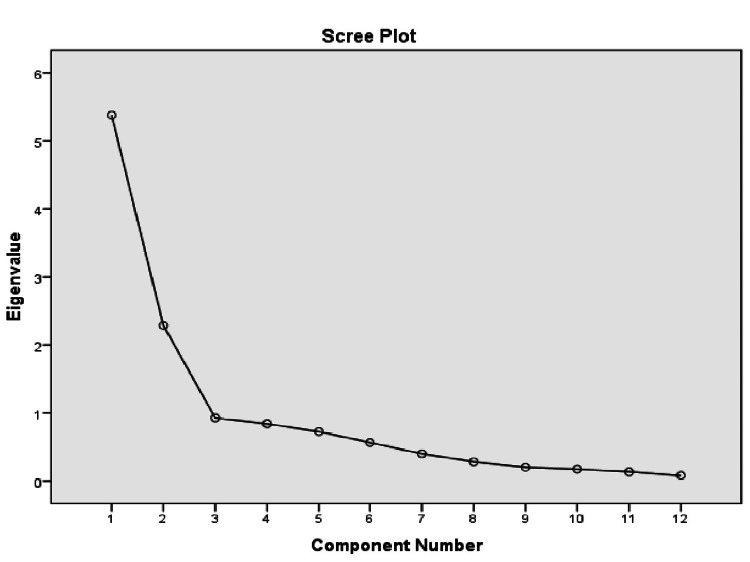
Scree plot representing the two extracted factors from principal component analysis and varimax rotation.

Principal component analysis and varimax rotation were used to extract the factors. Scree plot was obtained and assessed. Two components, Compliance and Hesitance, were formed on H-OHVS based on the scree plot elbow (Figure 1). The eigenvalues were greater than one, with Components 1 and 2 having eigenvalues of 5.382 and 2.287, respectively, accounting for 63.91% of the cumulative variance. The items with higher loading on any of the two factors were considered under that factor. Item loadings ranged from 0.536 to 0.948, resulting in non-exclusion of any item from the translated OHVS. Factor 1, labeled Compliant, comprised items 1, 3, 5, 7, 10, and 12, while Factor 2, labeled Hesitant, comprised items 2, 4, 6, 8, 9, and 11. The factor loading matrices of H-OHVS are reported in Table 4.

**Figure 2:**
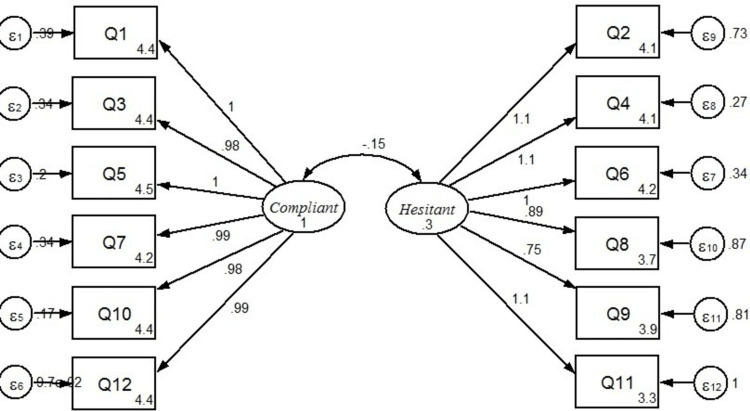
Two-factor confirmatory factor analysis representing the relationship between items and constructs. ε, measurement errors in each item

A structural equation modeling was performed using Stata v.16.1 as a tool to perform two-factor CFA to investigate the relationship between items and constructs. The maximum likelihood estimation approach was used. The components, Compliant and Hesitant, were considered as a latent construct of the related item - indicators (Figure 2). The latent constructs, Compliant and Hesitant, were allowed to co-vary in the model. The resulting model fit indices were RMSEA = 0.094 (90% confidence interval [CI] 0.078-0.110), CFI = 0.944, TLI = 0.931, and SRMR = 0.044. Three of the indices - CFI, TLI, and SRMR - presented adequate fit to the data based on the values obtained.

**Table 4:** Factor loading matrices of the Hindi version of the Oral Health Values Scale. Factor 1 = Compliant; Factor 2 = Hesitant. *Reverse-coded items, i.e., the positive items were rephrased negatively.

	Factor 1	Factor 2
Q1- It is essential for me to keep my natural teeth.	0.881	-0.148
Q2*- I often do not brush my teeth for a day or two when I am busy.	-0.089	0.638
Q3- My smile is an important part of my appearance.	0.885	-0.181
Q4*- Going to a dentist is not worth the cost to me.	-0.085	0.773
Q5- Flossing my teeth every day is a high priority for me.	0.922	-0.121
Q6*- I would rather get dentures than spend money to treat cavities or gum disease.	-0.054	0.747
Q7- I think it is important that my teeth and gums are a source of pride.	0.886	-0.103
Q8*- If I have a toothache, I prefer to wait and see if it will go away on its own before seeing a dentist.	-0.075	0.589
Q9*- I would not mind if I had to have a false tooth or dentures.	-0.088	0.536
Q10- I make sure I have dental floss available with me so I have it when I need it.	0.921	-0.118
Q11*- Going to the dentist is only important if my teeth or gums are bothering me.	-0.142	0.616
Q12- The condition of my teeth and gums is an important part of my overall health.	0.948	-0.086

Reliability coefficient

The overall Cronbach's alpha coefficient for H-OHVS (α = 0.868) presents an excellent internal consistency. The components, Compliant (α = 0.96) and Hesitant (α = 0.72), showed excellent and acceptable internal consistency, respectively.

## Discussion

This study aimed to assess the validity and reliability of the H-OHVS. The translation and adaptation of this tool can aid in evaluating the importance and values one gives to oral health. AAOS guidelines were followed to carry out the cross-cultural adaptation. Considerable efforts were also undertaken to adhere to stringent criteria while selecting the translators and experts. Furthermore, the participants were representative of both rural and urban populations, which is an important component of assessment, especially in a diverse country like India.

The analysis revealed that H-OHVS has common factors, and two components were formed in the principal component analysis. The translated tool was also reported to have adequate fit, as reported in the confirmatory analysis. H-OHVS has shown good reliability and validity results. However, it differs from the four components reported in the English [4] and the Romanian version of the OHVS [12].

The importance of oral health in India differs from the rest of the world, especially in terms of the lack of awareness and understanding of the importance of oral health. Studies in the past have even reported that the Indian population first seeks to resolve problems using self-short-term care and might eventually find some relief. Lesser demand for aesthetic treatment, prevailing myths related to dental treatment, the prevalence of unqualified practitioners primarily in rural areas, and local personalities serving as models of tobacco consumption and unkempt oral health [13], despite India progressing like never before, differentiate its oral health values from the world.

Previous reports have suggested that not even 100% of the population uses regular oral hygiene measures such as toothbrushes and toothpaste [14]. Flossing is not a common oral hygiene practice in India, and many studies in the past have reported regarding lack of awareness in the Indian population [15]. Understanding the importance and value of toothbrushing as an oral hygiene measure holds greater significance from an Indian perspective. The domain of flossing was thus replaced by toothbrushing, and the following items were subsequently modified during the translation and adaptation of the OHVS.

The final revisions were as follows: Question 2, "It is okay for me to miss a day or two of flossing when I am busy," was modified to "It is okay for me to miss a day or two of tooth brushing when I am busy." Question 5, "Flossing my teeth every day is a high priority for me," was modified to "Brushing my teeth every day is a high priority for me." Question 6, "I would rather get dentures than spend money to treat cavities or gum disease," was adjusted to "I would rather get dentures or false teeth than spend money to treat cavities or gum disease." Question 10, "I make sure I have dental floss available with me so I have it when I need it," was revised to "I make sure I have a toothbrush available with me so I have it when I need it."

Confirmatory factorial analysis supported the integral presence of all 12 items. Three indices presented adequate data fit. The indices fit reliability and validity analysis revealed that the findings are in line with those obtained in the validation of the English version. In contrast with the English version of the four-factor structure of the scale, only two factors of the scale were reported in H-OHVS. The reason could be attributed to the fact that no hierarchical model, as opposed to the American sample, exists in India. In the American sample, the appearance and retention subscale reported the highest mean scores followed by professional care and flossing. The H-OHVS further reported that the oral health value subscale in the Indian sample comprised only Compliant and Hesitant components. This further establishes the fact that there exist wide gaps in valuing oral health for appearance, retention of teeth, and other subscales, rather than the focus remaining on the two components.

Both the subscales, Compliant and Hesitant, revealed excellent and acceptable internal consistency. The overall scale also presented excellent internal consistency. In the original scale, only two subscales reported good internal consistency, whereas the other two reported reduced consistency. The reason for excellent internal consistency in H-OHVS could be related to an equal number of items in both subscales as opposed to a smaller number of items in subscales with reduced internal consistency in the original scale.

Limitations

Considerable efforts were undertaken to include participants truly representative of the Indian population by including them from different regions and socioeconomic statuses. However, the wide diversity of the Indian population and representativeness could not be specifically attributed. The data were less probabilistic, which can make the results less representative. This limitation provides the opportunity for further research, as future studies can target validation of this scale in different populations. One limitation that has been highlighted in previous studies too is related to the fact that data regarding the frequency of visits to a dentist were not collected. Thus, this scale can serve as a benchmark for the validity of OHVS and future research in different groups in the Indian population as well as in examination of different properties of OHVS.

## Conclusions

This study is the first of its kind to assess the psychometrical properties of H-OHVS. The study findings ascertain some degree of evidence in favor of this scale and establish the OHVS as a psychometrically sound measure. This scale can serve as a tool in conducting epidemiological research on oral health in the Indian population and in understanding how much the Indian population values oral health. This scale can aid in understanding and changing the perception of the importance of oral health education.

## Appendices

Appendix A

**Table 5:** Back-translated English version of the Oral Health Values Scale.

Question Number	Question
Question 1	It is essential for me to keep my natural teeth.
Question 2	I often do not brush my teeth for a day or two when I am busy
Question 3	My smile is essential to my looks.
Question 4	It is a waste for me to visit the dentist.
Question 5	I need to brush my teeth every day.
Question 6	Instead of wasting my money on getting my rotten teeth or gums treated, I would rather get dental implants or a denture.
Question 7	I consider my teeth and gums should be a source of pride to me.
Question 8	Whenever I have a toothache I prefer waiting for the pain to go away on its own before visiting the dentist.
Question 9	I don’t mind getting a denture implant or a denture.
Question 10	I make sure to carry toothpaste and a toothbrush with me.
Question 11	I only visit a dentist when I experience pain or uneasiness in my gums or teeth.
Question 12	My oral health is pertinent to my overall health.

Appendix B

**Table 6:** Hindi version of the Oral Health Values Scale.

प्रश्न 1	मेरे लिए अपने प्राकृतिक दांत रखना महत्वपूर्ण है।
प्रश्न 2	व्यस्त होने पर मेरे लिए एक या दो दिन दांतों की सफाई ना करना आम बात है।
प्रश्न 3	मेरी मुस्कान मेरे रूप का अह अहम् हिस्सा है।
प्रश्न 4	दन्त चिकित्सक के पास जाना मेरे लिए व्यर्थ है।
प्रश्न 5	हर दिन अपने दांतों की सफाई करना मेरे लिए सबसे ज़रूरी है।
प्रश्न 6	दांतों की सड़न या मसूड़े की बीमारी के इलाज के लिए पैसे खर्च करने के बजाय मैं नकली दांत या बत्तीसी बनवाना पसंद करूंगा।
प्रश्न 7	मुझे लगता है कि यह महत्वपूर्ण है कि मेरे दांत और मसूड़े मुझे गर्व महसूस करने का ज़रिया हैं।
प्रश्न 8	अगर मेरे दांत में दर्द है, तो मैं इंतजार करना पसंद करता हूं कि दंत चिकित्सक को दिखाने से पहले यह अपने आप दूर हो जाएगा या नहीं।
प्रश्न 9	मुझे कोई आपत्ति नहीं होगी अगर मुझे नकली दांत या बत्तीसी लगवाना पड़े।
प्रश्न 10	मैं सुनिश्चित करता हूं कि मेरे पास दन्त मंजन एवं ब्रश उपलब्ध रहता है।
प्रश्न 11	दंत चिकित्सक के पास जाना तभी जरूरी है जब मेरे दांत या मसूड़ों में तकलीफ हो रही हो।
प्रश्न 12	मेरे दांतों और मसूड़ों की स्थिति मेरे संपूर्ण स्वास्थ्य का एक महत्वपूर्ण हिस्सा है।
